# Shear Wave Elastography for Non-Invasive Assessment of Skin Involvement in Systemic Sclerosis

**DOI:** 10.3390/diagnostics16182905

**Published:** 2026-09-09

**Authors:** Xuesong Liu, Hanlin Yin, Wanyi Lin, Chenhan Jia, Fenglin Wu, Xinyu Li, Chunhua Wu, Ruru Guo, Liangjing Lu, Lixin Jiang

**Affiliations:** 1 Department of Ultrasound, Renji Hospital, Shanghai Jiao Tong University School of Medicine, 145 Middle Shandong Rd, Shanghai 200001, China; liuxuesong@renji.com (X.L.); wuchunhua@renji.com (C.W.); 2 Department of Rheumatology, Renji Hospital, Shanghai Jiao Tong University School of Medicine, 145 Middle Shandong Rd, Shanghai 200001, China; hanlinyin123@163.com (H.Y.); lin_wan_yi@163.com (W.L.); jnm123@sjtu.edu.cn (C.J.); wu_fenglin@sjtu.edu.cn (F.W.); xinyiliu221003@163.com (X.L.); loisen@163.com (R.G.)

**Keywords:** systemic sclerosis, shear wave elastography, skin fibrosis, high-frequency ultrasound, dermal thickness

## Abstract

**Background/Objectives**: Systemic sclerosis (SSc) is an autoimmune fibrosing disorder in which cutaneous involvement is a major manifestation. We investigated whether shear wave elastography (SWE) can distinguish SSc from healthy controls and examined how SWE relates to clinical and histological indices of skin fibrosis. **Methods**: The study included 78 patients with SSc and 30 healthy controls (HCs). High-frequency ultrasound (HFUS) quantified skin thickness and SWE quantified stiffness at the fingers, dorsal hands, and forearms. Ultrasound findings were examined in relation to the modified Rodnan Skin Score (mRSS). In 15 patients with diffuse cutaneous SSc (dcSSc), matched forearm biopsies were additionally analyzed for dermal thickness and collagen content. **Results**: Relative to HCs, patients with dcSSc had greater epidermal and dermal thickness at all examined sites, while patients with lcSSc had greater dermal thickness only at the fingers. Associations between dermal thickness and mRSS varied by site (Spearman ρ, 0.47–0.66). SWE values were higher in SSc than in HCs. The AUCs for distinguishing SSc from HCs were 0.820 at the fingers, 0.734 at the dorsal hands, and 0.760 at the forearms. Within the dcSSc biopsy subgroup, SWE correlated with collagen volume fraction (ρ = 0.68, *p* = 0.0068) and histological dermal thickness (ρ = 0.63, *p* = 0.0142). **Conclusions**: SWE enables quantitative non-invasive characterization of skin stiffness in SSc. Matched forearm histology in a small dcSSc subgroup provides preliminary evidence that the imaging measurements converge with tissue-level measures. Validation in larger independent cohorts is needed.

## 1. Introduction

Systemic sclerosis (SSc) is an autoimmune connective-tissue disease in which vasculopathy, immune dysregulation, and fibrosis produce heterogeneous involvement of the skin and internal organs [1,2]. Cutaneous fibrosis is a prominent feature of the disease, but its distribution and severity vary considerably among patients. Reliable quantification of skin involvement is therefore important for characterizing disease burden, yet remains difficult in routine clinical assessment [1].

Clinical evaluation of SSc skin involvement relies largely on the modified Rodnan Skin Score (mRSS), a semiquantitative palpation-based assessment performed at predefined body sites [3]. Although widely used, the mRSS is examiner dependent and may be less sensitive to subtle or mild abnormalities. Histopathology can directly characterize dermal architecture and fibrosis [4,5], but biopsy samples only a limited area and is not well suited to repeated evaluation because it is invasive. These limitations have prompted interest in quantitative, non-invasive approaches that can complement clinical examination.

High-frequency ultrasound (HFUS) and shear-wave elastography (SWE) offer two complementary ways to interrogate the skin non-invasively. HFUS depicts superficial tissue layers at high spatial resolution and permits measurement of epidermal and dermal thickness [6,7]. SWE, in contrast, estimates tissue mechanical stiffness, which can reflect fibrosis but is also affected by other tissue properties [8,9,10,11]. Most previous SSc studies have evaluated these modalities by comparing patients with healthy individuals, relating imaging findings to mRSS, or incorporating ultrasound into multimodal assessment [12,13,14]. Direct evidence relating SWE to anatomically matched histology remains limited and inconsistent. Further evaluation with site-matched tissue sampling and consideration of SSc subtype is therefore needed.

We therefore examined HFUS-derived skin thickness and SWE-derived stiffness as quantitative markers of skin involvement in SSc. Measurements were compared between patients with SSc and healthy controls, related to mRSS, and evaluated for their ability to discriminate SSc from healthy controls. In a dcSSc subgroup with matched-site forearm biopsies, we further examined whether ultrasound measurements were associated with histological dermal thickness and collagen deposition.

## 2. Materials and Methods

### 2.1. Study Population

We performed a single-center cross-sectional study within the Renji Scleroderma Longitudinal Cohort (Renji-SLOC), in accordance with the 2013 Declaration of Helsinki. Ethics approval was granted by the Ethics Committee of Renji Hospital, Shanghai Jiao Tong University School of Medicine (No. 2024-180; 1 January 2024), and written informed consent was obtained from every participant. Between January and December 2024, 78 patients with SSc and 30 healthy controls (HCs) were enrolled. Patients who met eligibility criteria during attendance at the rheumatology clinic were recruited consecutively; screening, exclusions, and final enrollment are summarized in Supplementary Figure S1. SSc classification followed the 2013 ACR/EULAR criteria [15]. Using the LeRoy classification [16], patients were categorized as diffuse cutaneous SSc (dcSSc) or limited cutaneous SSc (lcSSc) according to the distribution of skin involvement in relation to the elbows and knees. Participants were required to be older than 18 years. Clinical evaluation, mRSS, skin ultrasound, and laboratory testing were completed at enrollment, with histological assessment performed when applicable. Disease duration was defined from the first non-Raynaud’s symptom attributable to SSc. Exclusion criteria were another rheumatic disease, primary Raynaud’s phenomenon, skin or metabolic disease affecting the evaluated sites, and previous radiotherapy or chemotherapy.

### 2.2. mRSS and Assessment of Disease Activity

Two experienced rheumatologists independently assessed skin involvement with the mRSS while blinded to ultrasound findings. Seventeen anatomical sites were graded by palpation from 0 to 3, giving a possible total score of 0–51 [3,17]. mRSS and ultrasound examinations were completed during the same visit, between 12:00 PM and 4:30 PM, in a room maintained at 20–25 °C [18].

Disease activity was quantified with the preliminarily revised EUSTAR activity index [19]. The index assigns weighted contributions to patient-reported skin worsening (Δ-skin, 1.5), digital ulcers (1.5), tendon friction rubs (2.25), CRP > 1 mg/dL (2.25), DLCO < 70% predicted (1.0), and mRSS (1.5 points when >18; otherwise mRSS × 0.084). Scores of 2.5 or higher indicate active disease.

### 2.3. Skin Ultrasound

Ultrasound examinations were performed on a Canon Aplio i900 system (Canon Medical Systems, Otawara, Tochigi, Japan) using an 18 MHz transducer (i18LX5; 4–18 MHz bandwidth) and a 24 MHz transducer (i24LX8; 9–24 MHz bandwidth). Two sonographers, both with more than 5 years of experience in SWE and musculoskeletal ultrasound, acquired the examinations. Before enrollment began, both operators underwent protocol standardization training to reduce operator-related variation. A dedicated reliability substudy was not performed.

Anatomical sites were selected with reference to previous work examining skin stiffness at different locations [20]. Bilateral measurements were obtained at the middle fingers (dorsum of the proximal phalanx), dorsal hands (between the second and third metacarpophalangeal joints), and dorsal forearms (junction of the distal one-third and proximal two-thirds). Three consecutive measurements were averaged for each side, after which the left- and right-sided means were averaged to obtain one participant-level value for statistical analysis.

The 24 MHz transducer was used for epidermal and dermal thickness measurements. A 5 mm hydrogel stand-off pad was placed on the skin to provide a reproducible acoustic interface and limit compression [21,22]; the probe was kept perpendicular to the surface without visible tissue deformation. Epidermal thickness was measured from the superficial skin surface to the hyperechoic dermal–epidermal interface. Dermal thickness extended from this interface to the hyperechoic boundary between dermis and subcutaneous fat and was measured with the system’s electronic calipers. Thickness values were recorded in millimeters.

SWE acquisition followed the thickness examination and used the 18 MHz transducer. After removal of the hydrogel pad, ample coupling gel was applied so that probe contact could be kept minimal. The transducer was oriented perpendicular to the skin and held stationary without visible deformation until the elastography map was stable and homogeneous. A circular ROI 1 mm in diameter was positioned completely within the dermis, excluding visible vessels and artifacts. Young’s modulus was recorded in kPa with the superficial soft-tissue SWE preset (measurement range, 0–800 kPa). Before imaging, participants rested for 15–20 min in a 20–25 °C room and were examined in a relaxed seated or supine position with the upper limbs fully supported. Operators remained blinded to mRSS findings. Acquisitions showing artifacts or unstable elastography maps were discarded and repeated.

### 2.4. Skin Biopsy and Histopathological Analysis

Of the 28 patients with dcSSc, 15 consented separately to the optional invasive component and underwent a 3 mm forearm punch biopsy, using the general biopsy approach described in our previous SSc work [23]. Tissue was sampled on the day of ultrasound from the site marked during imaging. Paraformaldehyde-fixed, paraffin-embedded sections were stained with hematoxylin and eosin (H&E) for skin architecture and with Masson’s trichrome for dermal collagen assessment. Because biopsy participation was voluntary, this histology group constitutes a selected subset of the dcSSc cohort.

Slides were digitized at 20× magnification (0.5 μm/pixel) with a Leica Aperio whole-slide scanner. Calibrated whole-slide images were analyzed in ImageJ (version 1.54f, National Institutes of Health, Bethesda, MD, USA). On H&E sections, dermal thickness was defined from the dermal–epidermal junction to the dermal–subcutaneous interface. On Masson’s trichrome sections, collagen volume fraction (CVF) was calculated as the collagen-positive area divided by the dermal ROI area. Three positions were randomly selected on each section, and their measurements were averaged for analysis. Two experienced pathologists, blinded to ultrasound and clinical information, evaluated the histological measurements. For visual comparison, one anatomically concordant healthy-control skin specimen obtained during elective excision of an intradermal naevus was also available. This specimen was collected with informed consent under ethics approval No. 2024-180 and was not entered into quantitative histological correlation analyses. No adverse events related to biopsy occurred.

### 2.5. Statistical Analysis

Continuous data are summarized as mean ± SD or median (IQR), according to distribution, and categorical data as number (%). Distributional normality was checked with the Shapiro–Wilk test. Depending on data distribution, two independent groups were compared with Student’s t test or the Mann–Whitney U test. Non-normally distributed outcomes across three independent groups were analyzed with the Kruskal–Wallis test, followed by Dunn’s post hoc comparisons with Bonferroni adjustment. Spearman coefficients (ρ) and corresponding *p* values were obtained with the Hmisc package in R; correlation matrices were generated with ggcorrplot. Benjamini–Hochberg false-discovery-rate adjustment was applied separately to the two correlation families, with *q* < 0.05 defining significance. Because the histology analysis included only 15 patients, correlation *p* values were also evaluated by 80,000-iteration permutation testing to reduce dependence on asymptotic approximations. Permutation-based Mann–Whitney *p* values were likewise used for the exploratory analyses in Supplementary Tables S6 and S7. Ninety-five percent confidence intervals for Spearman correlations were calculated using Fisher z-transformation. Partial Spearman analyses of ultrasound-derived measurements versus mRSS adjusted for age, current corticosteroid dose (prednisone-equivalent mg/day, coded as 0 for patients not taking corticosteroids), and disease duration; variables were rank transformed before residualisation. ROC AUCs and their 95% CIs were estimated with the DeLong method, and cut-offs were chosen by maximizing the Youden index. These cut-offs were both derived and assessed in the same dataset and underwent neither internal nor external validation. No a priori sample-size calculation was undertaken. A G*Power sensitivity analysis (version 3.1.9.7) showed that *n* = 78, two-sided α = 0.05, and 80% power permit detection of a minimum Spearman correlation of |ρ| = 0.31. Statistical analyses used GraphPad Prism 9 and R 4.4.1. A two-sided *p* < 0.05 was considered significant for analyses not undergoing multiplicity correction.

## 3. Results

### 3.1. Baseline Demographic and Clinical Characteristics

The demographic and clinical characteristics of the study participants are presented in Table 1. The cohort comprised 78 patients with SSc, including 28 with dcSSc and 50 with lcSSc, and 30 HCs. The SSc and HC groups were similar in sex distribution (84.6% vs. 83.3% female, *p* = 0.8680) and age (53.8 ± 13.6 vs. 49.3 ± 15.9 years, *p* = 0.1490).

Among patients with SSc, the median disease duration was 21.0 months (IQR, 6.7–55.5 months), and 53/78 (67.9%) had a disease duration of ≤36 months. The most common autoantibodies were anti-Scl-70 (44.9%) and anti-CENP-B (37.2%). Raynaud’s phenomenon was present in 92.3% of patients, and interstitial lung disease (ILD) was the most frequent organ manifestation (62.8%). The median EUSTAR activity index was 1.87 (IQR, 0.34–3.00).

### 3.2. Ultrasound-Measured Increases in Skin Thickness and Stiffness in Patients with SSc

**Figure 1:**
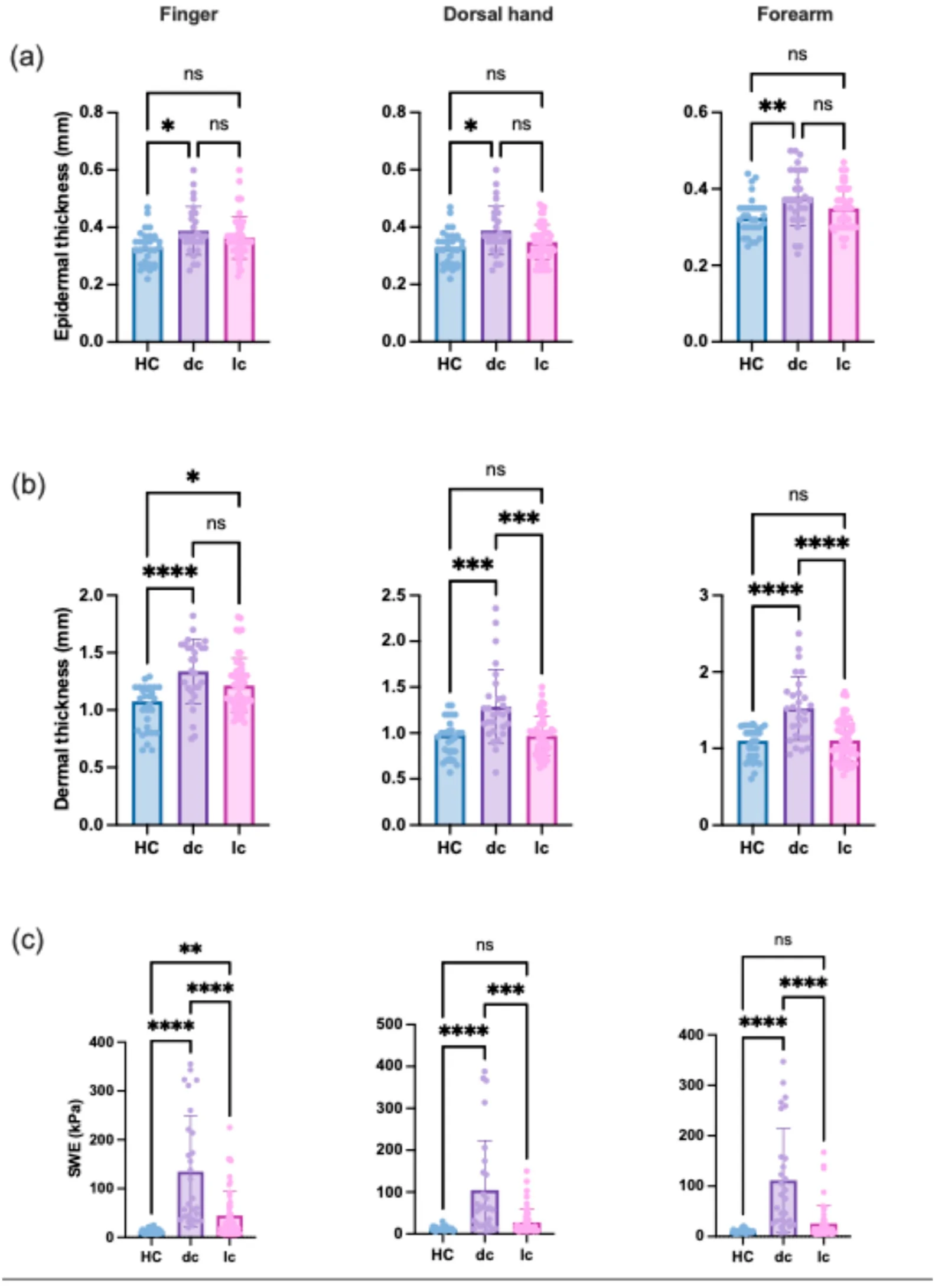
Skin thickness and stiffness assessed by ultrasound according to SSc subtype. Scatter plots show (**a**) epidermal thickness, (**b**) dermal thickness, and (**c**) dermal stiffness at the three anatomical sites in patients with dcSSc (*n* = 28), patients with lcSSc (*n* = 50), and HCs (*n* = 30). Horizontal lines represent medians. Three-group comparisons were performed using the Kruskal–Wallis test followed by Dunn’s post hoc test with Bonferroni correction. Significance levels: * *p* < 0.05, ** *p* < 0.01, *** *p* < 0.001, **** *p* < 0.0001.

In the subtype-stratified comparison, patients with dcSSc (*n* = 28) had greater epidermal and dermal thickness than HCs at the fingers, dorsal hands, and forearms. Patients with lcSSc (*n* = 50) showed greater dermal thickness than HCs at the fingers, whereas differences at the dorsal hands and forearms were not statistically significant. Dermal thickness at the dorsal hands and forearms was also greater in dcSSc than in lcSSc (Figure 1a,b). Descriptive statistics for these ultrasound-derived measures at each site are provided in Supplementary Table S1.

In the pooled analysis (Supplementary Table S8), patients with SSc had significantly greater epidermal thickness than HCs at all three sites (fingers: median 0.35 vs. 0.33 mm, *p* = 0.013; dorsal hands: 0.35 vs. 0.32 mm, *p* = 0.0019; forearms: 0.35 vs. 0.33 mm, *p* = 0.026), and significantly greater dermal thickness at the fingers (1.23 vs. 1.08 mm, *p* = 0.0034) and dorsal hands (1.01 vs. 0.98 mm, *p* = 0.042), but not at the forearms (1.20 vs. 1.20 mm, *p* = 0.207). Skin stiffness measured by SWE was significantly greater in patients with SSc than in HCs at all three sites (fingers: median 37.10 vs. 13.60 kPa, *p* < 0.0001; dorsal hands: 20.25 vs. 11.45 kPa, *p* = 0.0002; forearms: 20.55 vs. 9.55 kPa, *p* < 0.0001). Thus, pooled SSc–HC differences were significant in eight of nine site-specific comparisons, with the sole exception being forearm dermal thickness (*p* = 0.207).

SWE-derived skin stiffness also differed across the subtype-stratified groups. Patients with dcSSc had higher SWE values than both patients with lcSSc and HCs at all three anatomical sites. Patients with lcSSc had higher SWE values than HCs at the fingers, whereas the corresponding differences at the dorsal hands and forearms were not statistically significant. SWE values were higher in dcSSc than in lcSSc at all measured sites (Figure 1c). Descriptive statistics for these ultrasound-derived measures at each site are provided in Supplementary Table S1.

### 3.3. Correlations of Ultrasound Measurements with mRSS

**Figure 2:**
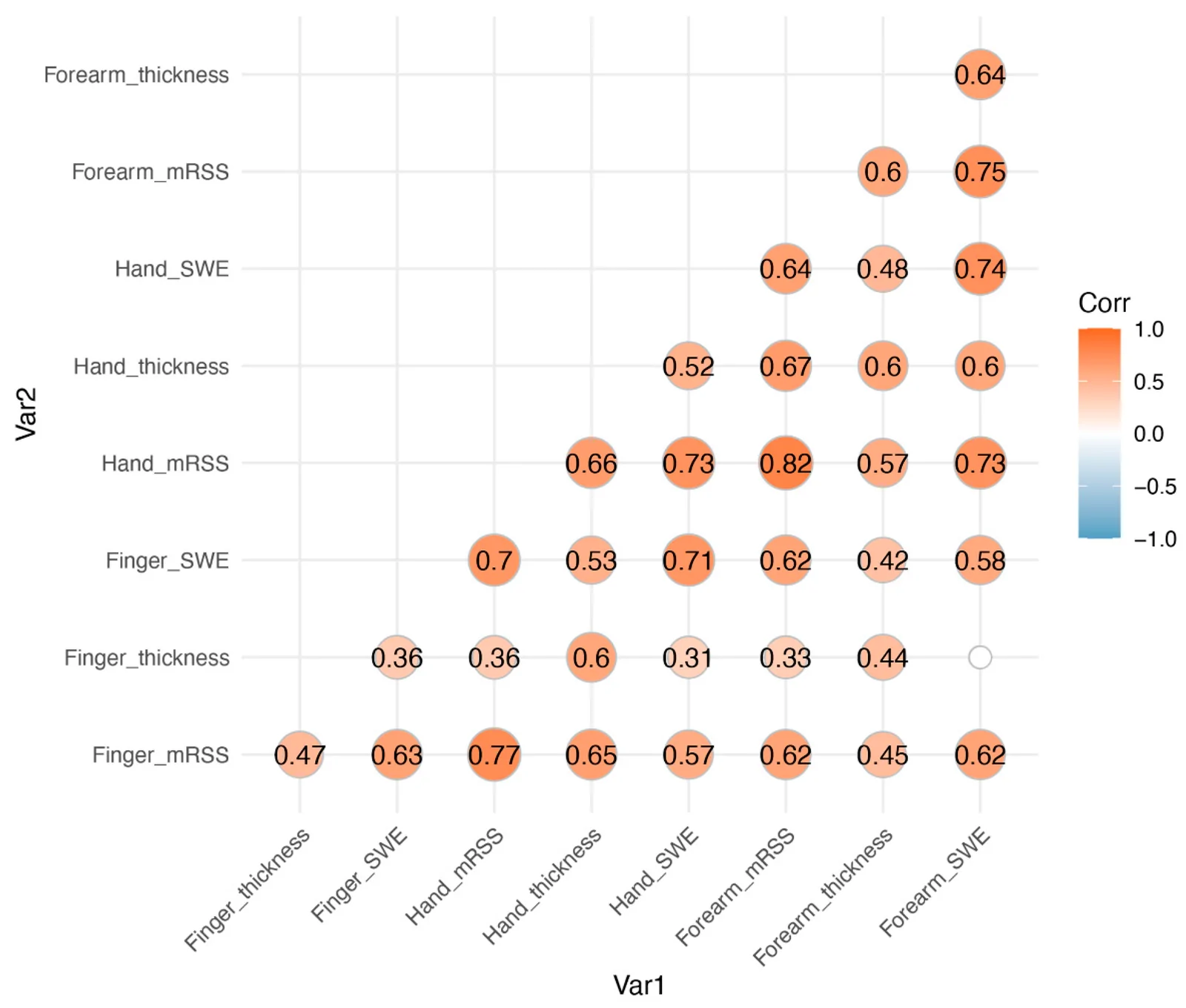
Correlation matrix of ultrasound parameters and mRSS in the SSc cohort (*n* = 78). Spearman’s rank correlations are shown for dermal thickness, SWE-derived skin stiffness, and mRSS across the three anatomical sites. For visual clarity, “thickness” in the matrix axis labels refers specifically to dermal thickness. Correlation coefficients (ρ) are displayed within the circles. Blank cells indicate nominally non-significant correlations (*p* ≥ 0.05); all coefficients and Benjamini–Hochberg q-values are reported in Supplementary Table S5.

As shown in Figure 2, SWE was positively correlated with mRSS across the measured sites. SWE-derived stiffness was positively associated with dermal thickness at all three sites, with the weakest association at the fingers (ρ = 0.365, *p* = 0.0010). The complete correlation coefficients, including non-significant results, are provided in Supplementary Table S5. After Benjamini–Hochberg correction, 35 of 36 pairwise correlations remained significant; the only non-significant association was between finger dermal thickness and forearm SWE stiffness (ρ = 0.206, *q* = 0.070). In partial Spearman analyses adjusted for age, current prednisone-equivalent corticosteroid dose, and disease duration, the associations of dermal thickness and SWE stiffness with site-matched mRSS remained significant at all three sites (partial ρ = 0.518–0.715; all *p* < 0.001; Supplementary Table S4).

### 3.4. Correlations Between Histological Findings and Ultrasound Measurements

**Figure 3:**
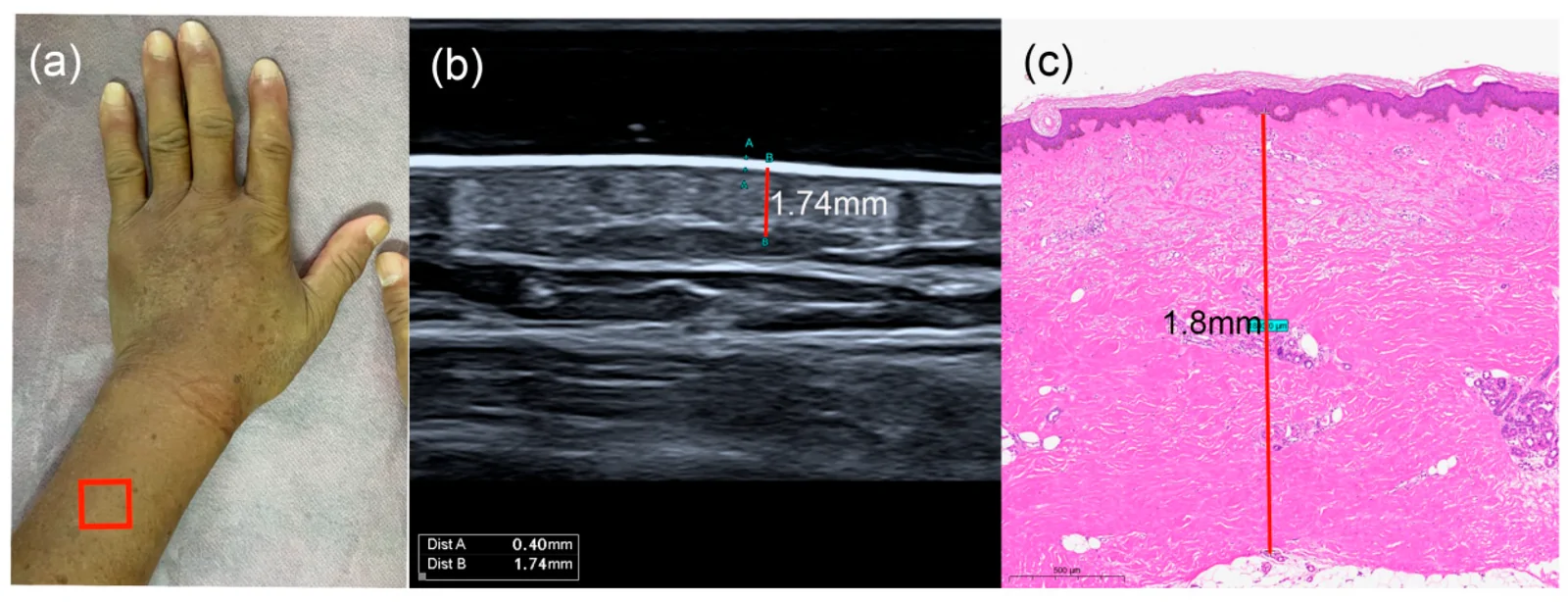
Representative images of a patient with dcSSc (overall biopsy cohort: *n* = 15). (**a**) Photograph of the forearm in a patient with dcSSc, with the red square indicating the site for ultrasonography and biopsy. (**b**) High-frequency grayscale ultrasound image with dermal thickness measurement. (**c**) H&E-stained skin section from the corresponding region, with dermal thickness measured for comparison.

Matched forearm histological sections were available from 15 patients with dcSSc who underwent the optional biopsy procedure. Descriptive statistics for the histological and matched ultrasound variables in this subgroup are provided in Supplementary Table S2. H&E-stained sections were compared with ultrasound-derived dermal thickness measurements from the corresponding anatomical site (Figure 3).

**Figure 4:**
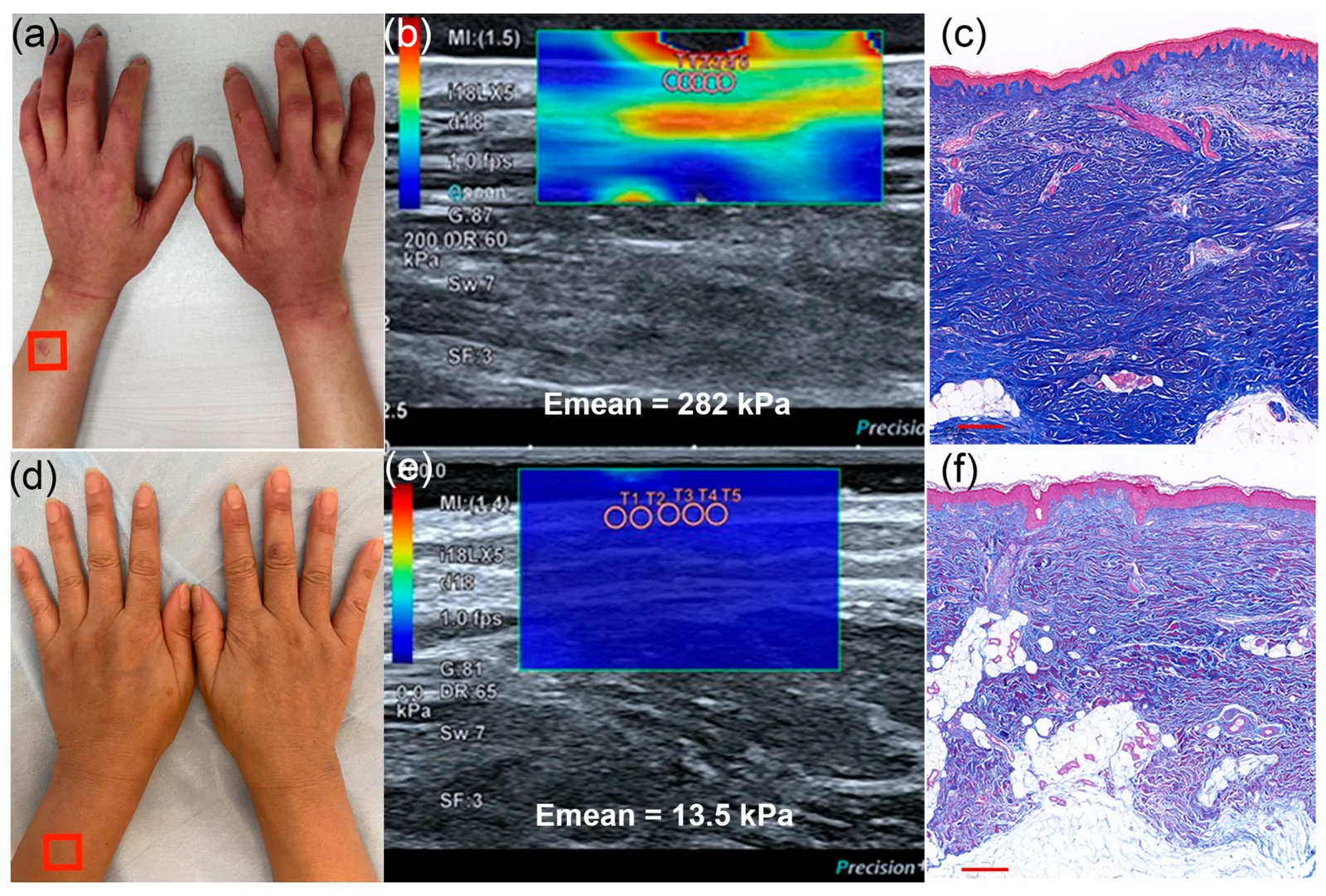
Representative comparative skin assessment in dcSSc and a healthy control. (**a**–**c**) Representative forearm photograph, SWE map, and Masson’s trichrome-stained section from a patient in the dcSSc biopsy cohort (*n* = 15). (**d**–**f**) Corresponding representative images from the single healthy control specimen available for visual comparison only. The healthy control specimen was not included in the quantitative histological correlation analyses. Scale bar = 200 μm.

Masson’s trichrome staining was used to quantify dermal collagen in the 15 dcSSc biopsy specimens and to examine its relationship with SWE measurements obtained at the corresponding forearm site. A representative healthy-control specimen is shown in Figure 4.

**Figure 5:**
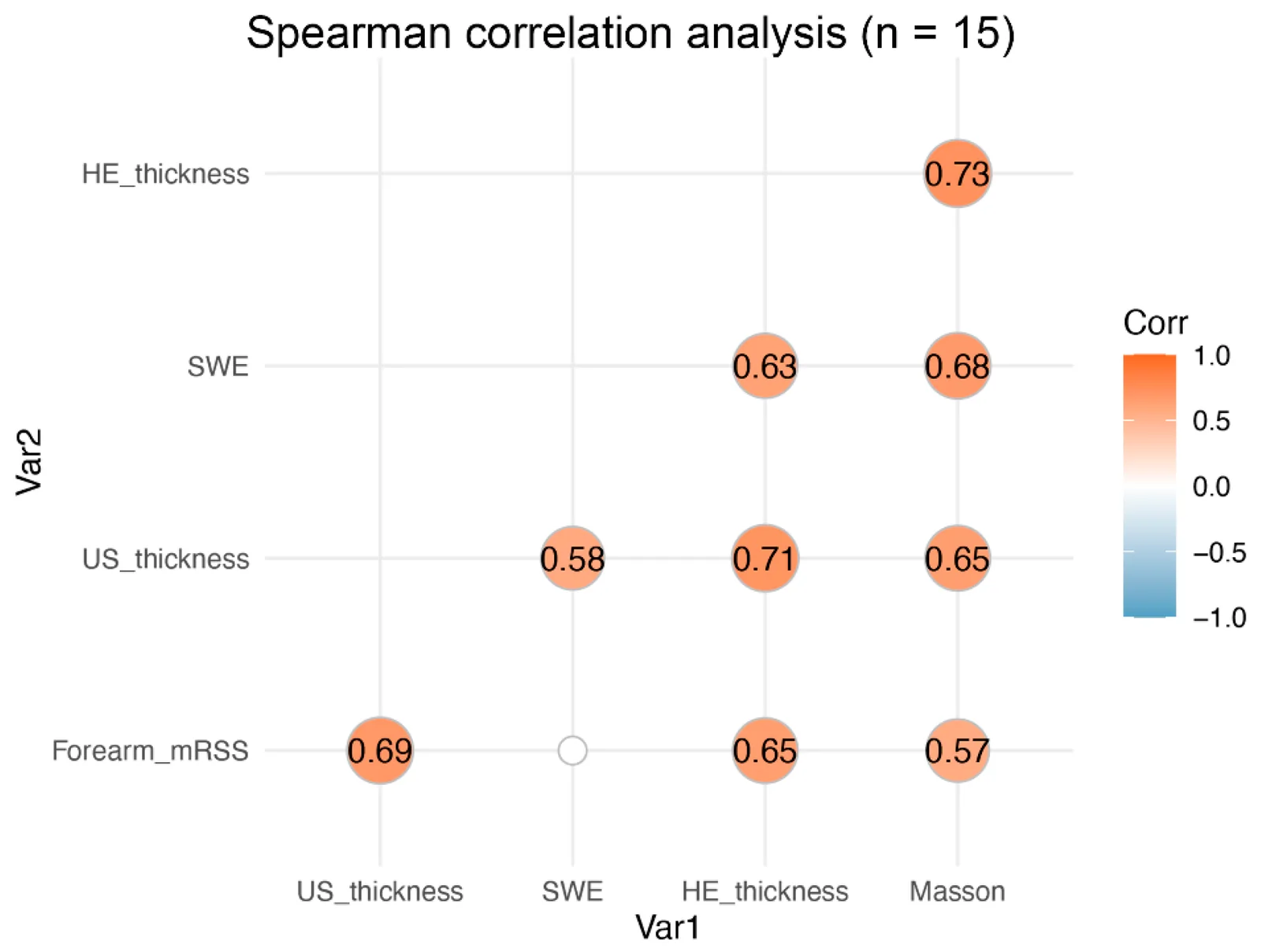
Correlations between histological, clinical, and ultrasound-based skin measurements in the dcSSc biopsy subgroup (*n* = 15). Spearman’s rank correlations are shown for histological dermal thickness (H&E), collagen volume fraction (Masson’s trichrome), mRSS, ultrasound-derived dermal thickness, and SWE-derived stiffness. Correlation coefficients (ρ) are displayed within the circles. Blank cells indicate nominally non-significant correlations (*p* ≥ 0.05); all coefficients, including non-significant values, are reported in Supplementary Table S5. Because the histology subgroup was small (*n* = 15), *p* values were additionally assessed by permutation testing (80,000 iterations); Benjamini–Hochberg q-values for the full set of correlations are reported in Supplementary Table S5.

In the 15-patient dcSSc biopsy subgroup, histological dermal thickness was positively correlated with ultrasound-measured dermal thickness (ρ = 0.71, 95% CI [0.31, 0.90], *p* = 0.0042). Collagen volume fraction (CVF) was positively correlated with SWE-derived stiffness (ρ = 0.68, 95% CI [0.26, 0.88], *p* = 0.0068) and with mRSS (ρ = 0.57, 95% CI [0.08, 0.84], *p* = 0.0319). SWE-derived stiffness was also positively correlated with histological dermal thickness (ρ = 0.63, 95% CI [0.17, 0.86], *p* = 0.0142) (Figure 5). Bland–Altman analysis demonstrated a systematic positive difference between histological and ultrasound-derived dermal thickness, with a mean bias of +0.57 mm and 95% limits of agreement of 0.11 to 1.03 mm (Supplementary Figure S2). Given the small biopsy subgroup and the number of correlations examined, these nominal *p* values are interpreted as exploratory; no superiority of the SWE–CVF correlation over the mRSS–CVF correlation is inferred. Complete correlation results, together with Benjamini–Hochberg-adjusted q-values calculated from permutation-based *p* values (80,000 iterations), are provided in Supplementary Table S5. After correction, nine of ten correlations remained significant; the SWE–CVF association remained significant (ρ = 0.679, *q* = 0.017), whereas the mRSS–SWE association did not (ρ = 0.444, *q* = 0.098).

### 3.5. Discrimination Between SSc and Healthy Controls Using SWE

**Figure 6:**
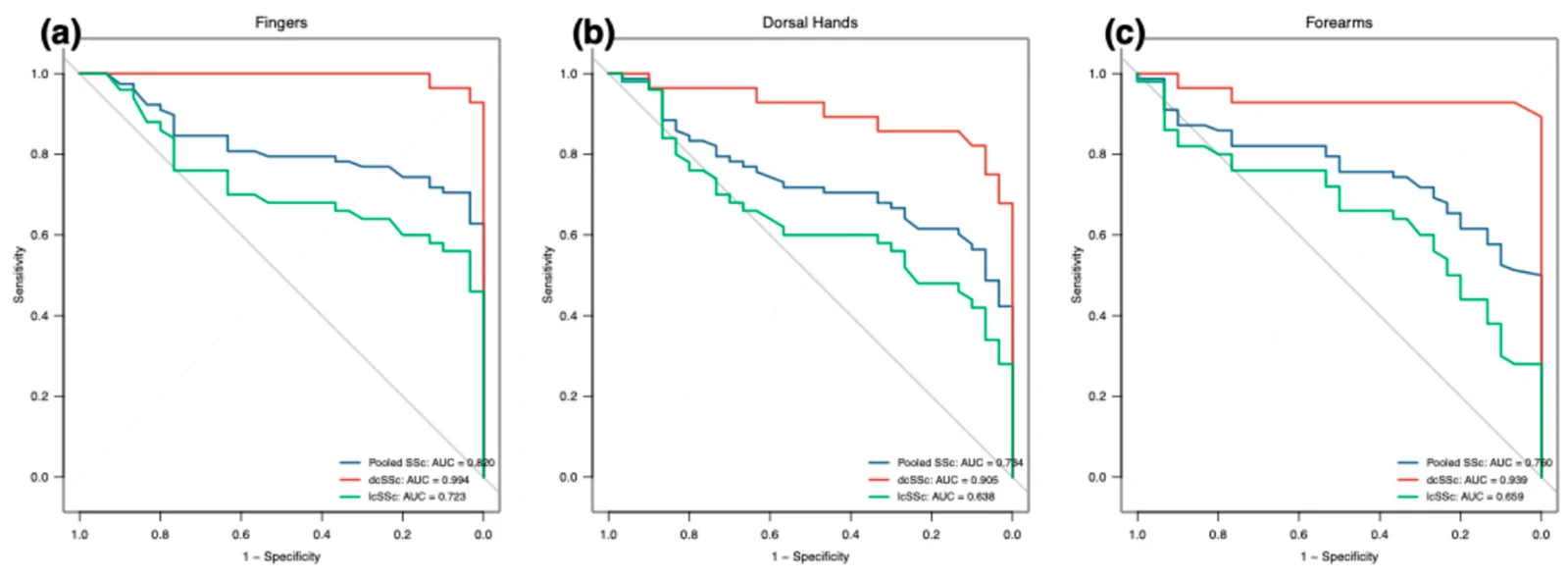
Receiver operating characteristic curves of shear wave elastography for discriminating systemic sclerosis from healthy controls according to disease subtype. ROC curves are shown for the (**a**) fingers, (**b**) dorsal hands, and (**c**) forearms. At each anatomical site, curves are presented for the pooled systemic sclerosis (SSc) cohort (*n* = 78), diffuse cutaneous SSc (dcSSc; *n* = 28), and limited cutaneous SSc (lcSSc; *n* = 50), each compared with healthy controls (HCs; *n* = 30). The corresponding AUCs for pooled SSc, dcSSc, and lcSSc were 0.820, 0.994, and 0.723 at the fingers; 0.734, 0.905, and 0.638 at the dorsal hands; and 0.760, 0.939, and 0.659 at the forearms, respectively. The grey diagonal line represents the reference line for no discrimination (AUC = 0.5). Detailed 95% confidence intervals, *p* values, optimal cut-off values, sensitivities, and specificities are provided in Supplementary Table S3.

ROC analysis was used to assess the ability of SWE-derived skin stiffness to discriminate patients with SSc from healthy controls at each anatomical site (Figure 6). At the finger site, SWE yielded an AUC of 0.820 (95% CI, 0.743–0.897; *p* < 0.0001); the Youden-index-derived cut-off of >22.45 kPa corresponded to a sensitivity of 70.5% and a specificity of 96.7%. At the dorsal hand, the AUC was 0.734 (95% CI, 0.642–0.826; *p* < 0.0001) with a cut-off of >19.20 kPa (sensitivity 56.4%, specificity 93.3%). At the forearm, the AUC was 0.760 (95% CI, 0.672–0.848; *p* < 0.0001) with a cut-off of >20.55 kPa (sensitivity 50.0%, specificity 100.0%) (Supplementary Table S3). The numerically highest AUC was observed at the fingers; however, the confidence intervals overlap and no formal between-site superiority is inferred. Because skin involvement differs markedly between disease subtypes, we additionally evaluated subtype-specific discrimination (Figure 6). In dcSSc (*n* = 28), SWE discriminated sharply from healthy controls at every site, with AUCs of 0.994 (95% CI, 0.983–1.000), 0.905 (95% CI, 0.819–0.991), and 0.939 (95% CI, 0.858–1.000) at the fingers, dorsal hands, and forearms, respectively (all *p* < 0.0001, DeLong). In lcSSc (*n* = 50), discriminatory performance was lower, with AUCs of 0.723 (95% CI, 0.612–0.834; *p* < 0.001), 0.638 (95% CI, 0.518–0.759; *p* = 0.025), and 0.659 (95% CI, 0.540–0.778; *p* = 0.009) at the fingers, dorsal hands, and forearms, respectively. These analyses indicate that the pooled AUCs are driven predominantly by the strong discrimination in dcSSc and that SWE’s overall diagnostic performance is strongly influenced by disease subtype (Supplementary Table S3). Because the cut-offs were derived and evaluated in the same cohort without bootstrap correction or independent validation, the reported sensitivity and specificity should be considered apparent (in-sample) performance estimates.

## 4. Discussion

Our findings indicate that HFUS and SWE capture complementary quantitative features of SSc skin involvement. Both thickness and stiffness differed between SSc and healthy-control skin, and their relationships with clinical skin scores varied across anatomical sites. The imaging measures may therefore add objective structural information to conventional clinical assessment.

The distribution of HFUS abnormalities differed by cutaneous subtype. In dcSSc, epidermal and dermal thickening extended across several anatomical sites, whereas in lcSSc the increase in dermal thickness was mainly confined to the fingers. This pattern accords with the clinical distinction between diffuse and limited cutaneous involvement. Herrick et al. [24] likewise described broader, more proximal skin involvement in dcSSc and predominantly distal disease in lcSSc. These observations emphasize that ultrasound findings should be interpreted in an anatomical site-specific context.

SWE measurements were also greater in SSc than in healthy controls. Earlier studies have supported the feasibility of SWE for SSc skin assessment through patient–control comparisons and associations with mRSS [8,25,26,27]. In our cohort, site-matched associations of dermal thickness and SWE stiffness with mRSS persisted after accounting for age, current corticosteroid exposure, and disease duration, making it unlikely that these measured factors alone explain the observed relationships. The exploratory analysis of clinically uninvolved skin, however, did not show higher SWE stiffness at dorsal-hand or forearm sites scored mRSS = 0 than at corresponding healthy-control sites. Within SSc, sites scored mRSS > 0 were stiffer than sites scored 0. Thus, these cross-sectional data do not demonstrate a distinct SWE abnormality at clinically uninvolved dorsal-hand or forearm sites. Diagnostic discrimination also depended strongly on disease subtype: separation from healthy controls was much greater for dcSSc than for lcSSc. Consequently, the pooled AUCs partly reflect the presence of patients with more extensive cutaneous disease. This spectrum effect limits extrapolation to differential-diagnostic settings, where patients may have milder SSc or non-SSc disorders that alter skin properties. The present AUCs and in-sample thresholds should therefore not be regarded as validated diagnostic performance for such settings. Although the finger had the numerically largest pooled AUC, overlapping confidence intervals do not establish superiority over the other sites. Differences from previously published AUCs may arise from variation in cohort composition, anatomical sampling, acquisition protocols, and elastography implementation. Cross-platform comparison is further complicated because some investigators report shear-wave speed rather than Young’s modulus.

Matched histology provided an additional, tissue-level perspective on the ultrasound findings. Within the dcSSc biopsy subgroup, forearm SWE was associated with CVF, and ultrasound-derived dermal thickness was associated with histological dermal thickness. Histopathology directly depicts dermal structure and fibrosis but, because it is invasive and spatially limited, is impractical for routine repeated assessment. Masson’s trichrome offers a practical morphometric estimate of collagen-rich fibrotic tissue, although the stain is not chemically specific for collagen and CVF should be interpreted accordingly. Our observations are broadly in line with Cai et al. [27]. Flower et al. [28] demonstrated good repeatability of HFUS and explored its relationship with dermal collagen, while Desai et al. [29] described moderate associations between ultrasound and histology. Chen et al. [30], by contrast, did not identify a significant relationship between ultrasound stiffness and histological measures. Heterogeneity in transducer frequency, ROI placement, sampling location, acquisition procedures, and histological processing may account for some of these differences. In the 15-patient biopsy subgroup, forearm SWE had a weaker, non-significant association with mRSS (ρ = 0.444, *q* = 0.098) than in the full SSc cohort (ρ = 0.749). The smaller sample and narrower severity range of this selected dcSSc subgroup reduce the ability of rank-based analyses to detect associations; this result should not be taken to imply an absence of a relationship between SWE and clinical skin involvement in dcSSc.

Bland–Altman analysis identified a systematic offset between the two methods, with histological dermal thickness exceeding the ultrasound measurement on average. Correlation between the measurements therefore does not imply that they can be used interchangeably. The techniques assess skin under fundamentally different conditions: ultrasound is acquired in vivo, whereas histological thickness is measured after excision, fixation, and tissue processing. Differences related to probe pressure, fixation or shrinkage, spatial resolution, and the choice of measurement boundaries may all contribute to the observed offset [31]. These factors need to be considered when comparing ultrasound and histological estimates of dermal thickness.

The study has several limitations. Its cross-sectional, single-center design and modest cohort size constrain generalizability, and histology was available only from a voluntary forearm-biopsy subgroup of 15 patients with dcSSc. Quantitative histology from healthy controls and disease-control groups was unavailable. Moreover, comparison with healthy volunteers creates a spectrum effect that may yield better discrimination than would be expected in clinically relevant differential-diagnostic populations. Although adjusted analyses accounted for age, current corticosteroid exposure, and disease duration, unmeasured confounding remains possible; BMI was not systematically recorded and could not be incorporated. SWE stiffness should not be viewed as fibrosis specific because non-fibrotic tissue properties can also influence the measurement. We did not obtain study-specific observer reliability data, and both HFUS and SWE depend on operator technique and acquisition protocol, including probe pressure and ROI placement. Measurements of Young’s modulus in thin dermal layers may also be affected by layer thickness and platform-specific behavior, potentially influencing stiffness–thickness associations and limiting the transferability of absolute thresholds. Finally, ROC cut-offs were derived from the same cohort in which they were evaluated and require independent validation before application to other populations or ultrasound systems. Multicenter and longitudinal studies are needed to define the clinical role of these techniques more clearly.

## 5. Conclusions

HFUS and SWE quantified differences in skin thickness and stiffness between SSc and healthy controls, with associations to clinical skin scores that depended on anatomical site. In the selected dcSSc biopsy subgroup, forearm imaging measurements also corresponded with histological measures of dermal fibrosis, providing preliminary cross-sectional evidence of convergent construct validity. These results support further evaluation of SWE as a complementary quantitative approach to skin assessment in SSc.

## Figures and Tables

**Table 1:** Demographic and clinical characteristics of SSc and healthy controls.

	Systemic Sclerosis (*n* = 78)	Healthy Controls (*n* = 30)	*p*
Age (yrs), mean ± SD	53.8 ± 13.6	49.3 ± 15.9	0.1490
Sex (Female)	66 (84.6%)	25 (83.3%)	0.8680
Disease duration (month), median and IQR	21.0 (6.7–55.5)	NA	NA
Short disease duration (≤36 months)	53 (67.9%)	NA	NA
mRSS, median (IQR)	7 (2–13)	NA	NA
lcSSc	4 (2–8)	NA	NA
dcSSc	17 (11–25)	NA	NA
SSc type			
lcSSc	50 (64.1%)	NA	NA
dcSSc	28 (35.9%)	NA	NA
Antibody			
Anti-Scl70	35 (44.9%)	NA	NA
Anti-CENP-B	29 (37.2%)	NA	NA
Anti-RNA-polymerase III	6 (7.7%)	NA	NA
Others	10 (12.8%)	NA	NA
Raynaud’s phenomenon	72 (92.3%)	NA	NA
ILD	49 (62.8%)	NA	NA
PAH	10 (12.8%)	NA	NA
Gastrointestinal symptoms	47 (60.3%)	NA	NA
Musculoskeletal symptoms	49 (62.8%)	NA	NA
Renal crisis	1 (1.3%)	NA	NA
Δ-skin	17 (21.8%)	NA	NA
Tendon friction rubs	3 (3.8%)	NA	NA
Digital ulcer	12 (15.4%)	NA	NA
DLCO predicted < 70%	34 (43.6%)	NA	NA
C-reactive protein > 1 mg/dL	4 (5.1%)	NA	NA
EUSTAR activity index, median and IQR	1.87 (0.34–3.00)	NA	NA
Treatments			
Corticosteroids	47 (60.3%)	NA	NA
csDMARDs	49 (62.8%)	NA	NA
Mycophenolate	32 (41.0%)	NA	NA
Methotrexate	4 (5.1%)	NA	NA
Cyclophosphamide	4 (5.1%)	NA	NA
Hydroxychloroquine	17 (21.8%)	NA	NA
Biological agents	3 (3.8%)	NA	NA
Tocilizumab	3 (3.8%)	NA	NA
Antifibrotic therapy	11 (14.1%)	NA	NA
Nintedanib	8 (10.3%)	NA	NA
Pirfenidone	3 (3.8%)	NA	NA

Abbreviations: dcSSc, diffuse cutaneous systemic sclerosis; lcSSc, limited cutaneous systemic sclerosis; mRSS, modified Rodnan skin score; CENP-B, centromere protein B; ILD, interstitial lung disease; PAH, pulmonary arterial hypertension; DLCO%, carbon monoxide diffusing capacity of the lungs; Δ-skin, patient assessed worsening during the previous month; csDMARD, conventional synthetic disease-modifying anti-rheumatic drugs. Categorical variables are expressed as number (%). Continuous parameters following a normal distribution are expressed as mean ± SD; other parameters are expressed as median (IQR).

## Data Availability

Data available on request.

## Supplementary Materials

The following supporting information can be downloaded at: https://www.mdpi.com/article/10.3390/diagnostics16182905/s1, Figure S1: Patient enrollment flowchart; Figure S2: Bland–Altman plot of ultrasound-derived and histological dermal thickness in the dcSSc biopsy subgroup (*n* = 15); Table S1: Descriptive statistics of clinical and ultrasound variables for Spearman correlation analysis in the SSc cohort; Table S2: Descriptive statistics of histological and ultrasound variables for Spearman correlation analysis in the dcSSc biopsy subgroup; Table S3: Diagnostic performance of SWE for distinguishing SSc from healthy controls (pooled and by subtype); Table S4: Partial Spearman correlations between ultrasound measurements and mRSS after adjustment for age, current corticosteroid dose, and disease duration (*n* = 78 patients with SSc); Table S5: Benjamini–Hochberg adjusted *q*-values for the reported correlation analyses Figure 2 (Family A) and Figure 5 (Family B); Table S6: Subclinical skin-stiffness analysis: SWE at mRSS = 0 sites versus healthy-control sites; Supplementary Table S7: Exploratory disease-duration analysis: Ultrasound measurements and mRSS by short (≤36 months) versus long (>36 months) disease duration; Table S8: Pooled comparison of ultrasound-derived skin measurements between patients with systemic sclerosis(SSc) and healthy controls (HCs).

## Abbreviations

The following abbreviations are used in this manuscript:

ACR	American College of Rheumatology
AUC	Area under the curve
CENP-B	Centromere protein B
csDMARD	Conventional synthetic disease-modifying anti-rheumatic drug
CVF	Collagen volume fraction
dcSSc	Diffuse cutaneous systemic sclerosis
DLCO	Diffusing capacity of the lungs for carbon monoxide
EULAR	European League Against Rheumatism
H&E	Hematoxylin and eosin
HCs	Healthy controls
HFUS	High-frequency ultrasound
ILD	Interstitial lung disease
lcSSc	Limited cutaneous systemic sclerosis
mRSS	Modified Rodnan Skin Score
PAH	Pulmonary arterial hypertension
Renji-SLOC	Renji Scleroderma Longitudinal Cohort
ROC	Receiver operating characteristic
ROI	Region of interest
SSc	Systemic sclerosis
SWE	Shear-wave elastography
