# Supplementary material for: Shear Wave Elastography for Non-Invasive Assessment of Skin Involvement in Systemic Sclerosis
Source: Diagnostics (Basel). 2026 Sep 9;16(18):2905. doi: 10.3390/diagnostics16182905 (PMC13605320; doi:10.3390/diagnostics16182905)
Supplement: Supplementary file 1 [file diagnostics-16-02905-s001.zip › diagnostics-4457962-supplementary.pdf]

Supplementary Materials

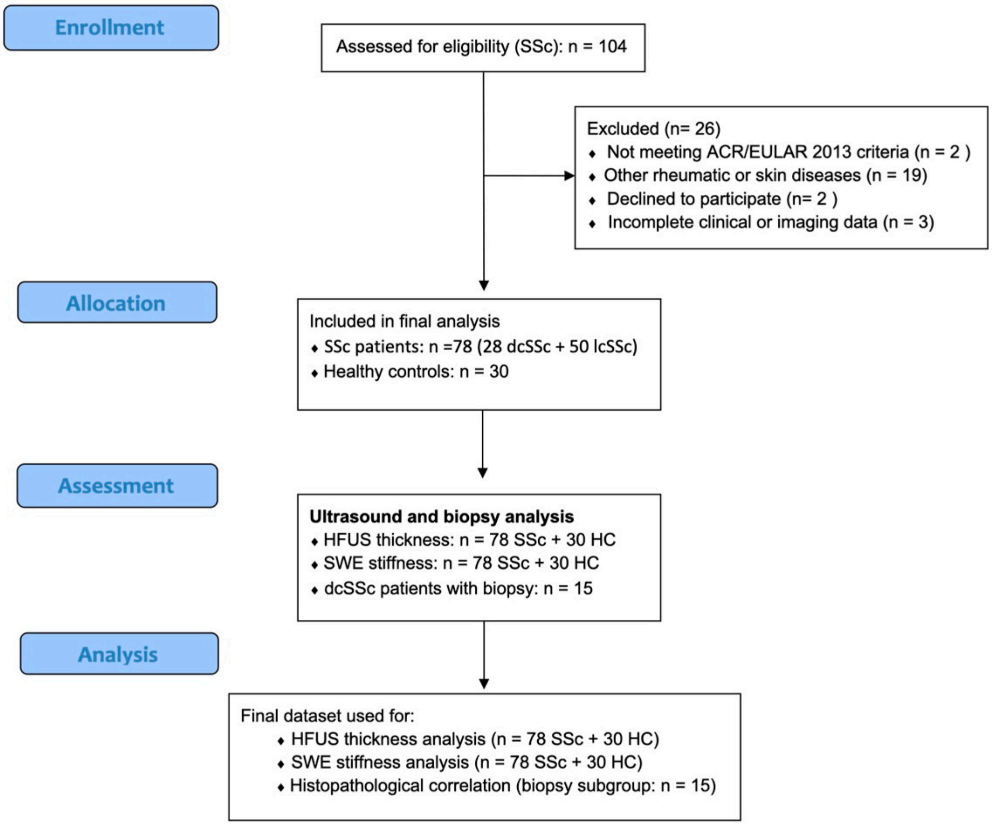

Supplementary Figure S1. Patient enrollment flowchart. The diagram illustrates the screening, exclusion, and final allocation of participants in the study. Of 104 individuals with systemic sclerosis (SSc) assessed for eligibility, 26 were excluded. The final cohort included 78 patients with SSc and 30 healthy controls (HCs), all of whom underwent ultrasound assessments. A subgroup of 15 patients with diffuse cutaneous SSc (dcSSc) underwent additional skin biopsy for histopathological correlation.

Supplementary Table S1. Descriptive statistics of clinical and ultrasound variables for Spearman correlation analysis in the SSc cohort (*n* = 78).

**Variable (*n* = 78)**	**Median (IQR)**
Finger mRSS	2 (1–3)
Finger dermal thickness (mm)	1.23 (1.07–1.44)
Finger SWE (kPa)	37.1 (18.5–104.0)
Hand mRSS	1 (0–2)
Hand dermal thickness (mm)	1.01 (0.87–1.24)
Hand SWE (kPa)	20.25 (10.5–60.3)
Forearm mRSS	0 (0–1)
Forearm dermal thickness (mm)	1.20 (0.97–1.48)
Forearm SWE (kPa)	20.55 (10.7–64.8)

Values are presented as median (IQR).

Dermal thickness measured by HFUS; SWE stiffness given in kPa.

Abbreviations: mRSS, modified Rodnan Skin Score; SWE, shear wave elastography; IQR, interquartile range.

Supplementary Table S2. Descriptive statistics of histological and ultrasound variables for Spearman correlation analysis in the dcSSc biopsy subgroup (*n* = 15).

**Variable (*n* = 15)**	**Median (IQR)**
Forearm mRSS	2 (1.5–2.5)
US thickness (mm)	1.6 (1.5–1.9)
SWE (kPa)	123.0 (53.2–213.0)
H&E thickness (mm)	2.24 (2.06–2.51)
Masson (collagen volume fraction, %)	52.27 (41.80–68.17)

Values are presented as median (IQR).

Collagen volume fraction (CVF) was quantified from Masson's trichrome-stained histological sections.

Abbreviations: mRSS, modified Rodnan Skin Score; SWE, shear wave elastography; US, ultrasound; HE, hematoxylin and eosin; IQR, interquartile range. Dermal thickness measured by high-frequency ultrasound is denoted as US thickness; dermal thickness measured on H&E-stained histological sections is denoted as HE thickness.

Supplementary Table S3. Diagnostic performance of SWE for discriminating SSc from healthy controls.

	AUC, 95% CI	*p*	Cut-off value (kPa)	Sensitivity (%)	Specificity (%)
Pooled SSc — Fingers	0.820 [0.743–0.897]	<0.0001	>22.45	70.5%	96.7%
Pooled SSc — Dorsal hand	0.734 [0.642–0.826]	<0.0001	>19.20	56.4%	93.3%
Pooled SSc — Forearm	0.760 [0.672–0.848]	<0.0001	>20.55	50.0%	100.0%
dcSSc — Fingers	0.994 [0.983–1.000]	<0.0001	>23.50	96.4%	96.7%
dcSSc — Dorsal hand	0.905 [0.819–0.991]	<0.0001	>19.45	82.1%	93.3%
dcSSc — Forearm	0.939 [0.858–1.000]	<0.0001	>21.70	89.3%	100.0%
lcSSc — Fingers	0.723 [0.612–0.834]	<0.001	>22.45	56.0%	96.7%
lcSSc — Dorsal hand	0.638 [0.518–0.759]	0.025	>19.20	42.0%	93.3%
lcSSc — Forearm	0.659 [0.540–0.778]	0.009	>11.35	60.0%	73.3%

AUC and 95% CI computed by the DeLong method. Cut-off values selected by the Youden index. Sensitivity and specificity are apparent (in-sample) estimates; cut-offs were derived and evaluated in the same cohort without independent validation. *N* (HC) = 30 for all comparisons. dcSSc: diffuse cutaneous SSc (*n* = 28); lcSSc: limited cutaneous SSc (*n* = 50). Abbreviations: AUC, area under the curve; CI, confidence interval; Se, sensitivity; Sp, specificity.

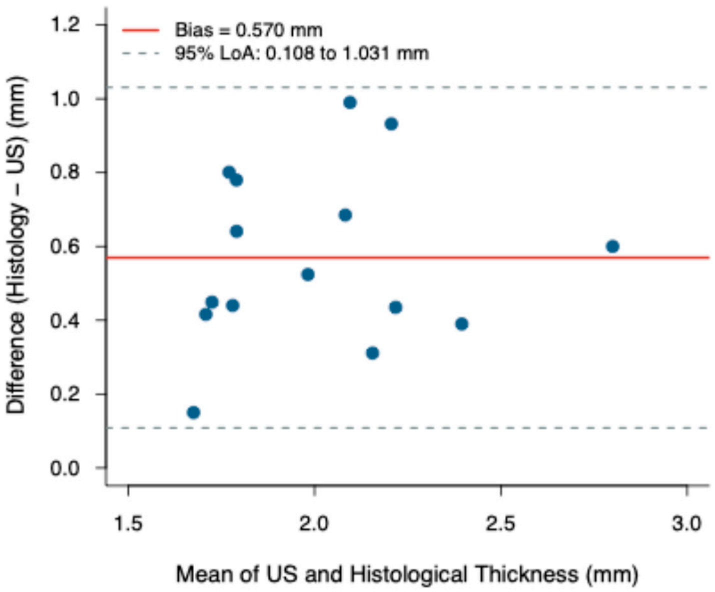

Supplementary Figure S2. Bland–Altman plot of ultrasound-derived and histological dermal thickness in the dcSSc biopsy subgroup (*n* = 15). The x-axis shows the mean of the two measurements; the y-axis shows their difference (histology minus US). The solid red line represents the mean bias (+0.57 mm); dashed grey lines represent the 95% limits of agreement (0.11 to 1.03 mm).

Supplementary Table S4. Partial Spearman correlations between ultrasound-derived skin measurements and the modified Rodnan skin score (mRSS), adjusted for age, current corticosteroid dose, and disease duration (*n* = 78 patients with SSc).

Site – Measure	Crude ρ	Adjusted ρ	Adjusted p
Finger – dermal thickness	0.475	0.518	2.28×10^{-6}
Finger – SWE stiffness	0.628	0.608	7.14×10^{-9}
Dorsal hand – dermal thickness	0.663	0.651	2.63×10^{-10}
Dorsal hand – SWE stiffness	0.734	0.715	5.80×10^{-13}
Forearm – dermal thickness	0.602	0.619	3.13×10^{-9}
Forearm – SWE stiffness	0.749	0.709	1.05×10^{-12}

Partial Spearman correlations were adjusted for age, current corticosteroid dose (prednisone-equivalent mg/day; coded as 0 mg/day for patients not receiving corticosteroids), and disease duration (months). Variables were rank-transformed before residualisation. All six adjusted correlations remained significant (all $P < 0.001$). SWE, shear-wave elastography.

Supplementary Table S5. Benjamini–Hochberg adjusted *q*-values for the correlation analyses in Figure 2 (Family A) and Figure 5 (Family B).

Family A — Figure 2 correlation matrix (n = 78, m = 36 pairs). *P*-values from *t*-approximation to Spearman null distribution.

**Variable 1**	**Variable 2**	**Spearman ρ**	**BH q**
Finger dermal thickness	Finger SWE stiffness	0.365	1.1×10^{-3}
Finger dermal thickness	Finger mRSS	0.475	1.4×10^{-5}

Finger dermal thickness	Hand dermal thickness	0.600	1.2×10^{-8}
Finger dermal thickness	Hand SWE stiffness	0.314	5.3×10^{-3}
Finger dermal thickness	Hand mRSS	0.361	1.3×10^{-3}
Finger dermal thickness	Forearm dermal thickness	0.440	6.7×10^{-5}
Finger dermal thickness	Forearm SWE stiffness	0.206	0.070 (NS)
Finger dermal thickness	Forearm mRSS	0.328	3.6×10^{-3}
Finger SWE stiffness	Finger mRSS	0.628	1.9×10^{-9}
Finger SWE stiffness	Hand dermal thickness	0.533	7.3×10^{-7}
Finger SWE stiffness	Hand SWE stiffness	0.715	1.0×10^{-12}
Finger SWE stiffness	Hand mRSS	0.695	7.5×10^{-12}
Finger SWE stiffness	Forearm dermal thickness	0.417	1.7×10^{-4}
Finger SWE stiffness	Forearm SWE stiffness	0.578	4.9×10^{-8}
Finger SWE stiffness	Forearm mRSS	0.616	4.1×10^{-9}
Finger mRSS	Hand dermal thickness	0.654	2.8×10^{-10}
Finger mRSS	Hand SWE stiffness	0.575	5.5×10^{-8}
Finger mRSS	Hand mRSS	0.772	$< 10^{-15}$
Finger mRSS	Forearm dermal thickness	0.452	4.1×10^{-5}
Finger mRSS	Forearm SWE stiffness	0.617	4.1×10^{-9}
Finger mRSS	Forearm mRSS	0.616	4.1×10^{-9}
Hand dermal thickness	Hand SWE stiffness	0.516	1.8×10^{-6}
Hand dermal thickness	Hand mRSS	0.663	1.4×10^{-10}
Hand dermal thickness	Forearm dermal thickness	0.598	1.3×10^{-8}
Hand dermal thickness	Forearm SWE stiffness	0.600	1.2×10^{-8}
Hand dermal thickness	Forearm mRSS	0.669	8.7×10^{-11}
Hand SWE stiffness	Hand mRSS	0.734	1.5×10^{-13}
Hand SWE stiffness	Forearm dermal thickness	0.477	1.4×10^{-5}
Hand SWE stiffness	Forearm SWE stiffness	0.737	1.3×10^{-13}
Hand SWE stiffness	Forearm mRSS	0.641	7.8×10^{-10}
Hand mRSS	Forearm dermal thickness	0.575	5.5×10^{-8}
Hand mRSS	Forearm SWE stiffness	0.726	3.3×10^{-13}
Hand mRSS	Forearm mRSS	0.817	$< 10^{-15}$
Forearm dermal thickness	Forearm SWE stiffness	0.640	7.8×10^{-10}
Forearm dermal thickness	Forearm mRSS	0.602	1.1×10^{-8}
Forearm SWE stiffness	Forearm mRSS	0.749	3.8×10^{-14}

35/36 pairs significant at $q < 0.05$. Sole non-significant pair: Finger dermal thickness vs Forearm SWE stiffness ($q = 0.07$, marked NS).

Family B — Figure 5 correlation matrix ($n = 15$ dcSSc biopsy subgroup, $m = 10$ pairs). BH-adjusted q-values were calculated from permutation-based p values (80,000 iterations).

**Variable 1**	**Variable 2**	**Spearman ρ**	**BH q**
H&E thickness	CVF (Masson)	0.731	0.016
H&E thickness	mRSS	0.645	0.019
H&E thickness	US thickness	0.706	0.016
H&E thickness	SWE stiffness	0.627	0.021

CVF (Masson)	mRSS	0.570	0.032
CVF (Masson)	US thickness	0.652	0.019
CVF (Masson)	SWE stiffness	0.679	0.017
mRSS	US thickness	0.689	0.016
mRSS	SWE stiffness	0.444	0.098 (NS)
US thickness	SWE stiffness	0.584	0.031

9/10 pairs significant at $q < 0.05$. Sole non-significant pair: mRSS vs SWE stiffness ($q = 0.098$, marked NS). CVF, collagen volume fraction; H&E, haematoxylin and eosin; US, ultrasound.

Supplementary Table S6. Subclinical skin-stiffness analysis: SWE stiffness at mRSS = 0 sites versus healthy-control sites.

SSc sites scored as mRSS = 0 were compared with healthy-control (HC) sites at each anatomical site (finger excluded: only 6 finger sites with mRSS = 0).

Site	SSc mRSS=0, *n*	Median SWE (kPa)	HC, *n* / Median SWE (kPa)	*p* (Mann–Whitney, permutation)
Dorsal hand	35	11.10	30 / 11.45	0.72
Forearm	47	12.00	30 / 9.55	0.079

Within-SSc: mRSS = 0 vs mRSS > 0 sites:

Site	mRSS=0, *n*	mRSS>0, *n*	*p* (Mann–Whitney)
Dorsal hand	35	43	1.3×10^{-8}
Forearm	47	31	1.0×10^{-11}

SWE stiffness at mRSS = 0 sites did not differ significantly from healthy-control sites at either anatomical site.

Supplementary Table S7. Exploratory disease-duration analysis: ultrasound measurements and mRSS by short (≤36 months) versus long (>36 months) disease duration.

Patients dichotomised at 36 months ($n = 53$ short, $n = 25$ long). Mann–Whitney U test with permutation-based P-values.

Measure	Short (≤36 m), median	Long (>36 m), median	*p*
Finger – dermal thickness (mm)	1.30	1.10	0.04
Finger – SWE stiffness (kPa)	48.0	23.0	0.18
Finger – mRSS	3	2	0.60
Dorsal hand – dermal thickness (mm)	1.04	0.99	0.51
Dorsal hand – SWE stiffness (kPa)	33.2	15.0	0.13
Dorsal hand – mRSS	2	0	0.37
Forearm – dermal thickness (mm)	1.29	1.14	0.44
Forearm – SWE stiffness (kPa)	28.0	18.5	0.50
Forearm – mRSS	0	0	0.36

SWE stiffness did not differ significantly between groups at any site (all $p \geq 0.13$). The only nominally significant

comparison was finger dermal thickness (p = 0.04). mRSS did not differ between groups (all P ≥ 0.36). No multiplicity adjustment was applied across these nine exploratory comparisons; therefore, the isolated nominal p = 0.04 finding should not be interpreted as confirmatory evidence of a disease-duration effect.

Supplementary Table S8. Pooled comparison of ultrasound-derived skin measurements between patients with systemic sclerosis (SSc) and healthy controls (HCs).

Patients with SSc (*n* = 78) were compared with healthy controls (*n* = 30) at each anatomical site using the Mann–Whitney U test. Values are presented as median (IQR). Epidermal and dermal thickness measured by high-frequency ultrasound; SWE stiffness given in kPa.

**Measure**	**Site**	**SSc (*n* = 78), median (IQR)**	**HC (*n* = 30), median (IQR)**	***p***
Epidermal thickness (mm)	Fingers	0.35 (0.32–0.40)	0.33 (0.27–0.37)	0.013
Epidermal thickness (mm)	Dorsal hand	0.35 (0.32–0.40)	0.32 (0.30–0.35)	0.0019
Epidermal thickness (mm)	Forearm	0.35 (0.30–0.40)	0.33 (0.30–0.35)	0.026
Dermal thickness (mm)	Fingers	1.23 (1.07–1.44)	1.08 (0.80–1.20)	0.0034
Dermal thickness (mm)	Dorsal hand	1.01 (0.87–1.24)	0.98 (0.80–1.03)	0.042
Dermal thickness (mm)	Forearm	1.20 (0.97–1.48)	1.20 (0.90–1.30)	0.207
SWE stiffness (kPa)	Fingers	37.10 (18.50–104.00)	13.60 (10.00–17.57)	<0.0001
SWE stiffness (kPa)	Dorsal hand	20.25 (10.50–60.30)	11.45 (9.03–14.68)	0.0002
SWE stiffness (kPa)	Forearm	20.55 (10.70–64.80)	9.55 (7.90–11.82)	<0.0001

All three epidermal-thickness comparisons, all three SWE-stiffness comparisons, and finger and dorsal-hand dermal-thickness comparisons were significantly greater in patients with SSc than in HCs. Forearm dermal thickness did not differ significantly between groups (*p* = 0.207). Abbreviations: SSc, systemic sclerosis; HC, healthy control; SWE, shear-wave elastography; IQR, interquartile range.
